# Peptide YY Is Critical for Acylethanolamine Receptor Gpr119-Induced Activation of Gastrointestinal Mucosal Responses

**DOI:** 10.1016/j.cmet.2010.04.014

**Published:** 2010-06-09

**Authors:** Helen M. Cox, Iain R. Tough, Anne-Marie Woolston, Lei Zhang, Amy D. Nguyen, Amanda Sainsbury, Herbert Herzog

**Affiliations:** 1 King's College London, Wolfson Centre for Age-Related Diseases, Guy's Campus, London SE1 1UL, UK; 2 Neuroscience Program, Garvan Institute of Medical Research, 384 Victoria Street, Darlinghurst, Sydney, New South Wales 2010, Australia; 3 School of Medical Sciences, University of New South Wales, Sydney, New South Wales 2052, Australia; 4 Faculty of Medicine, University of New South Wales, Sydney, New South Wales 2052, Australia

**Keywords:** HUMDISEASE

## Abstract

Peptide YY (PYY) is released following food intake and regulates intestinal function and glucose homeostasis, but the mechanisms underpinning these processes are unclear. Enteroendocrine L cells contain PYY and express the acylethanolamine receptor, Gpr119. Here, we show that Gpr119 activation inhibited epithelial electrolyte secretion in human and mouse colon in a glucose-sensitive manner. Endogenous PYY selectively mediated these effects, since *PYY*^−/−^ mice showed no Gpr119 response, but responses were observed in *NPY*^−/−^ mice. Importantly, Gpr119 responses in wild-type (WT) mouse tissue and human colon were abolished by Y_1_ receptor antagonism, but were not enhanced by dipeptidylpeptidase IV blockade, indicating that PYY processing to PYY(3-36) was not important. In addition, Gpr119 agonism reduced glycemic excursions after oral glucose delivery to WT mice but not *PYY*^−/−^ mice. Taken together, these data demonstrate a previously unrecognized role of PYY in mediating intestinal Gpr119 activity and an associated function in controlling glucose tolerance.

## Highlights

► Endogenous PYY, but not NPY, mediates Gpr119 effects in human and mouse colon mucosa ► The action of endogenous PYY is mediated specifically via epithelial Y_1_ receptors ► Apical and basolateral Gpr119 responses are glucose sensitive ► Gpr119 agonism reduced glycemia after oral glucose in WT but not *PYY*^−/−^ mice

## Introduction

One of the major roles for intestine-derived peptides is the coordination of digestion with nutrient and electrolyte absorption. In addition, several of these peptides, such as glucagon-like peptide (GLP)-1 and GLP-2, act as incretins, mediating effects on nutrient uptake via augmented insulin release from pancreatic β cells (Drucker, 2005). Furthermore, gut peptides, including peptide YY (PYY), pancreatic polypeptide (PP), and GLP-1, signal satiety to the brain (Gardiner et al., 2008). Enteroendocrine L cells located predominantly in the distal ileum and colon of human and rodent intestine (Böttcher et al., 1984; Arantes and Nogueira, 1997) are the primary source of PYY, which is coreleased following food intake with proglucagon products, GLP-1 and GLP-2 (Gardiner et al., 2008).

Gastrointestinal (GI) function is regulated by enteric nerves, and neuropeptide Y (NPY) is an inhibitory neurotransmitter expressed in secretomotor neurons of the submucosal plexi (Mongardi Fantaguzzi et al., 2009). Together with PP and the dipeptidylpeptidase IV (DPP-IV)-cleaved products NPY(3-36) and PYY(3-36) (Mentlein et al., 1993), NPY and PYY exert a range of inhibitory activities, such as slowing gastric emptying, reducing intestinal anion and electrolyte secretion (Playford et al., 1990; Cox and Tough, 2002), and slowing intestinal motility, which collectively promote nutrient absorption. Modulation of GI functions also has important effects on food intake, energy expenditure, and glucose homeostasis by influencing the delivery of nutrients and gut hormones to the circulation.

PYY, PYY(3-36), NPY, and NPY(3-36) are prominent intestinal peptides that exert their inhibitory actions via different Y receptors. Notably, the antisecretory mucosal mechanisms by which these peptides exert their effects are the same in human and mouse colon, with Y_1_ receptor-mediated responses being solely epithelial, while Y_2_-mediated effects are neuronal in origin (Cox and Tough, 2002; Hyland et al., 2003; Cox, 2007). Anatomical and functional studies have shown that Y_1_ receptors are targeted to basolateral epithelial membranes (Mannon et al., 1999; Cox and Tough, 2002) and would therefore be activated by endogenous PYY or NPY released into the subepithelial area. Use of selective Y_1_ and Y_2_ receptor antagonists together with peptide null mice have allowed us to link endogenous PYY and NPY with their cognate receptors. We have shown that Y_1_-activated intestinal antisecretory effects are predominantly PYY mediated, while NPY preferentially stimulates neuronal Y_2_-mediated mucosal responses (Hyland et al., 2003; Tough et al., 2006; Cox, 2008).

PYY and proglucagon-derived peptides are copackaged in enteroendocrine L cells (Böttcher et al., 1984) that can be activated by a range of lumenal nutrients such as fatty acids of different lengths (Anini et al., 1999; Hirasawa et al., 2005); however, the mechanisms that underpin these processes have not been characterized in native tissues. Recently, it has been suggested that GI chemosensation is mediated by several unrelated G protein-coupled receptors (GPCRs), including Gpr119, Gpr120, and Gpr40 (Engelstoft et al., 2008). In particular, the expression pattern of Gpr119 is very similar to that of PYY/GLP-1 containing L cells (Chu et al., 2008), suggesting that Gpr119 stimulation could cause significant PYY-related responses as well as GLP-1-mediated effects in the colon and elsewhere. The endogenous Gpr119 ligand, oleoylethanolamide (OEA), has been shown to reduce food intake and weight gain (Overton et al., 2006) and to increase GLP-1 release from L cells in vitro and in vivo (Ahrén et al., 2004; Reimann et al., 2008). Additionally, Gpr119 agonism has been shown to improve glucose tolerance in association with enhanced glucose-induced circulating insulin concentrations (Overton et al., 2008). Since GLP-1 and PYY are copackaged (Böttcher et al., 1984) and coreleased from L cells and both peptides have effects on intestinal function and glucose homeostasis (Boey et al., 2007; Overton et al., 2008), it is likely that PYY is also important in mediating Gpr119 responses.

The primary aims of this study were therefore to identify the mechanisms by which endogenous PYY mediated Gpr119 activity in intact colonic tissue and if so, whether these altered epithelial electrolyte secretion and glucose tolerance. To achieve these aims, we utilized selective Y receptor antagonists together with specific transgenic mouse models and human colon mucosa. A further aim was to establish whether DPP-IV inhibition altered Gpr119-activated colonic responses. DPP-IV inhibitors are clinically proven antidiabetics that elevate plasma GLP-1 concentrations by prolonging the half-life of this and other peptides. In turn, this improves glycemia in type 2 diabetes (Ahrén et al., 2004), in part by prolonging insulin release (Demuth et al., 2005) and increasing insulin sensitivity together with a beneficial glucagon-lowering capacity (Ahrén, 2009). However, the broad substrate specificity of DPP-IV could also reduce the conversion rate of PYY to PYY(3-36) and particularly of NPY to NPY(3-36) (Mentlein et al., 1993; Lambeir et al., 2008), which could have adverse effects on other physiological functions. In fact, GI disturbance has been described as a side effect of current antidiabetic therapy based on DPP-IV inhibition (sitagliptin), while constipation is noted in some patients taking another DPP-IV inhibitor, vildagliptin (Lauster et al., 2007). It was therefore of interest to determine whether selective DPP-IV inhibition amplified Gpr119-activated colonic responses by prolonging the functional half-life of endogenous PYY and/or NPY.

## Results

In order to compare responses from the different null mice and human tissue, we first established the basal electrophysiological parameters of colonic mucosae from mice of each genotype used in this study. The results for wild-type (WT), *PYY*^−/−^, *NPY*^−/−^, and double knockout (*NPYPYY*^−/−^) colonic mucosae were similar, while human colonic data were in accord with previously published data (Cox and Tough, 2002; Tough et al., 2006) (Table S1). Epithelial vasoactive intestinal polypeptide (VIP) secretory responses are a consequence of G_αs_-coupled VPAC receptor stimulation that results in lumenally directed Cl^−^ secretion, and this anion movement is measured as an increase in short-circuit current (I_sc_). Reductions in I_sc_, termed antisecretory responses here, result for example from PYY or NPY stimulating epithelial G_αi_-coupled Y_1_ receptors, reducing cAMP-mediated Cl^−^ secretion and attenuating I_sc_ levels as a consequence (Cox et al., 1988). Such antisecretory effects were measured to three Y receptor agonists (chosen to preferentially stimulate Y_1_, Y_2_, or Y_4_ receptors), and the response sizes were the same in WT, single, and double peptide knockout colon (Figure S1). Of the genotypes used, only *PYY*^−/−^ mice showed increased body weight, as seen previously with the same knockout (Boey et al., 2006) and a different *PYY*^−/−^ mouse (Wortley et al., 2007) (Table S1). Thus, the ablation of PYY, NPY, or both peptides did not alter the sensitivity of colonic preparations to VIP or to subsequent Y agonists, and epithelial secretory and antisecretory pathways were unaltered.

### PYY Expression Is Unchanged in *NPY*^−/−^ Tissue Compared with WT Colon

**Figure 1:**
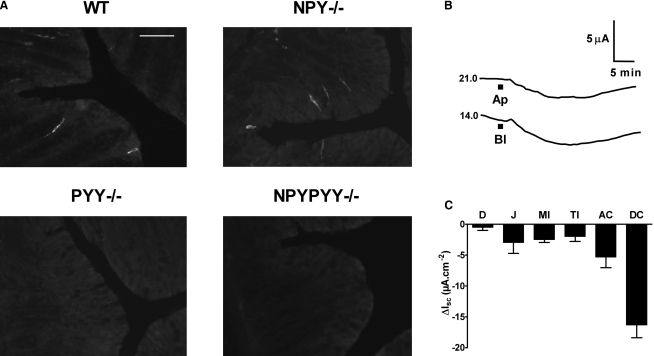
PYY-Positive L Cells in WT and *NPY*^−/−^ Mouse Colon and Gpr119 Responses in WT Mouse Intestinal Mucosa (A) Immunohistochemical localization showing distinct PYY-containing L cells in WT and *NPY*^−/−^ mucosae, but not in *PYY*^−/−^ or *NPYPYY*^−/−^ mouse descending colon. Scale bar, 20 μm throughout. (B) Example Gpr119 responses to apical (Ap) or basolateral (Bl) PSN632408 (10 μM). The basal I_sc_ levels are shown to the left of each trace, and mucosal area was 0.14 cm^2^. (C) Tissue sensitivity to apical PSN632408 (10 μM) in mouse mucosae from duodenum (D), jejunum (J), mid and terminal ileum (MI, TI), and ascending (AC) and descending colon (DC). Data are the mean − SEM from 3–14 observations. Background information, including basal electrophysiological parameters, is presented in Table S1. The pharmacology of selected Y agonist responses in WT versus null mouse colon mucosae are compared in Figure S1, with the patterns of NPY immunoreactivity in WT and null tissues presented in Figure S2.

To establish whether deletion of the NPY or PYY gene altered the expression pattern of the remaining peptide gene, colonic tissue was analyzed by immunohistochemistry. As shown in Figure 1A, PYY-positive L cells were observed throughout the colonic mucosa with the same frequency in WT (55.0 ± 4.6 cells/section) and *NPY*^−/−^ descending colon (56.7 ± 6.8 cells/section), and as expected, they were absent from *PYY*^−/−^ or *NPYPYY*^−/−^ tissues. In the descending colon, chromogranin-positive L cells were present with a similar frequency in each genotype (data not shown). The frequency and extent of intramural nerve PGP9.5 labeling was also similar across the genotypes (for example, WT and *NPYPYY*^−/−^ labeling, Figure S2). As expected, NPY-positive innervation was absent from *NPYPYY*^−/−^ (Figure S2) and *NPY*^−/−^ intestine (data not shown). Taken together with the consistent Y agonist sensitivities observed across the genotypes, these predicted patterns of immunolabeling confirm the lack of significant compensatory changes in peptide null colon, making them appropriate models for functional investigations.

### Apical and Basolateral Activation of Gpr119 Reduces I_sc_ in WT Mouse Tissue

Direct stimulation of enteroendocrine cells by nutrients provides a general sensing mechanism that depends crucially on the presence of different GPCRs (Engelstoft et al., 2008). Prominent among these is Gpr119, which is stimulated preferentially by lipid amides such as endogenous OEA (Overton et al., 2006). As the expression pattern of Gpr119 in the GI tract closely resembles that of PYY-expressing L cells, we chose to activate Gpr119 receptors using the small molecule agonist, PSN632408, which is less lipophilic and more selective for Gpr119 than OEA. Overton et al. (2006) showed that PSN632408 (at 10 μM), when tested against a panel of 107 GPCRs, channels, and transporters including peroxisome proliferator-activated receptor (PPAR)α and transient receptor potential cation channel V1 (TRPV1), inhibited ≤30% of binding or function. In WT and *NPY*^−/−^ colon mucosa, PSN632408 and OEA (10 μM) responses were insensitive to pretreatment with the PPARα antagonist, GW6471 (1 μM), and the TRPV1-desensitizing stimulus, capsaicin (2 μM) (Figures S3A–S3C).

In WT colonic mucosa, PSN632408 mimicked the antisecretory effects of PYY, causing long-lasting reductions in I_sc_ (Figure 1B). As Y_1_ receptors are trafficked selectively to epithelial basolateral domains (Mannon et al., 1999; Cox and Tough, 2002), we set out to establish whether Gpr119 responses were also polarized. As clearly shown in Figure 1B, they were not; PSN632408 addition to apical or basolateral colonic surfaces reduced the I_sc_ to similar levels and within a similar time frame. Importantly, these antisecretory responses were essentially identical to those of exogenous PYY and NPY in the same tissue (Cox et al., 2001; Hyland et al., 2003), suggesting a common pathway. A clear regional variation in Gpr119 sensitivity was also observed in WT mouse GI tract (Figure 1C), with apical PSN632408 responses being greatest in the descending colon and least in duodenal mucosa. This regional Gpr119 sensitivity correlates with the expression levels of Gpr119 mRNA (Chu et al., 2008) and L cell distribution, both of which are lowest in the proximal small intestine and highest in the descending colon of the mouse (Arantes and Nogueira, 1997) and human GI tract (Böttcher et al., 1984). A similar increasing sensitivity to exogenous PYY and NPY has also been described from the small to the large intestine of the mouse (Cox et al., 2001). It is important to note that PSN632408 (10 μM) had no effect on colonic smooth muscle activity in WT colon, nor were there alterations in contractile activity to PYY, PYY(3-36), NPY, or PP in the knockout models compared with WT tissue (data not shown), and thus we investigated mucosal functions further.

### Gpr119 Stimulation with PSN632408 Leads to Y_1_ Receptor Activation in Mouse Colon

**Figure 2:**
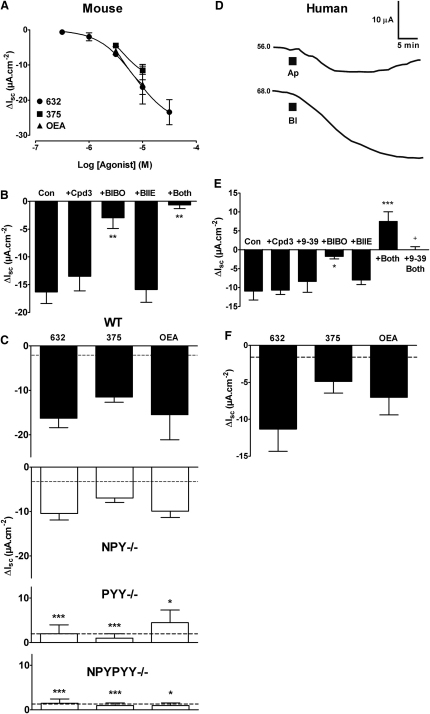
Colonic Responses to Gpr119 Agonists Are Y_1_ Receptor Mediated in Mouse and Human Colon Mucosae (A) Concentration response curve for apical PSN632408 (632, n = 3–14), with two concentration data points only for apical PSN375963 (375, n = 4–5) and OEA (n = 4–5) in WT mouse colon mucosa. Values are the mean ±SEM throughout. (B) Apical PSN632408 (10 μM) responses after pretreatment with either compound 3 (+Cpd 3, 1 μM) or the Y_1_ antagonist, BIBO3304 (+BIBO, 300 nM), or Y_2_ antagonism with BIIE0246 (+BIIE, 1 μM) alone or together with BIBO3304 (+Both). Data groups (mean − SEM) are compared with control PSN632408 responses (Con). ^∗∗^p ≤ 0.01. (C) Three different agonists (all apical, 10 μM) stimulate Gpr119 responses in WT colon (n = 5 or 14) and *NPY*^−/−^ (n = 3–4), but not *PYY*^−/−^ colon (n = 3–4) or *NPYPYY*^−/−^ colon (n = 4). Dashed lines show the mean vehicle control values (n = 3–4). Agonist responses in peptide knockouts are compared with WT responses. ^∗^p ≤ 0.05, ^∗∗∗^p ≤ 0.001. (D) Representative Gpr119 responses in human colon mucosa to apical (Ap) or basolateral (Bl) addition of PSN632408 (10 μM). Basal I_sc_ values are shown to the left of each trace, and the mucosal area was 0.64 cm^2^. (E) PSN632408 (10 μM) sensitivity to pretreatments with the DPP-IV inhibitor, compound 3 (+Cpd3, 1 μM), GLP-1 antagonist exendin(9-39) (+9-39, 1 μM), Y_1_ receptor antagonist BIBO3304 (+BIBO, 300 nM), Y_2_ antagonist BIIE0246 alone (+BIIE, 1 μM), Y_2_ antagonist BIIE0246 together with BIBO3304 (+Both), and BIIE0246 together with BIBO3304 and exendin(9-39) (+9-39 Both, n = 3–5). Comparisons are made with control PSN632408 responses (Con). ^∗^p ≤ 0.05, ^∗∗∗^p ≤ 0.001. (F) Single concentration (10 μM) effects of apical PSN632408 (632), PSN375963 (375), or OEA (all n = 4) with the mean vehicle controls (dashed line) in normal human colon mucosa. Table S1 includes basal electrophysiological parameters for mouse and human colon mucosal preparations used in this study. Figures S3A–S3C show that PSN632408 and OEA responses are insensitive to blockade of PPARα receptors and TRPV1 desensitization by capsaicin in WT and *NPY*^−/−^ colon.

To establish the link between Gpr119 and endogenous PYY or NPY function in colon mucosa, we tested PSN632408 in the absence or presence of selective Y receptor antagonists. First, however, we determined the potency of apical PSN632408. The consequent sustained reductions in I_sc_ exhibited an EC_50_ of 5.7 μM (2.2–14.6 μM) (Figure 2A) in WT mouse colon, consistent with a previous measure of potency at mouse Gpr119 receptors (7.9 ± 0.7 μM) (Overton et al., 2006). The agonist-response profile was likely to be bell shaped, because 100 μM apical PSN632408 reduced I_sc_ by only −7.9 μA/cm^2^, indicating desensitization. The proposed endogenous Gpr119 ligand, OEA, and another small molecule agonist, PSN375963 (Overton et al., 2006), reduced I_sc_ levels to the same degree as PSN632408 in WT colon (Figure 2A), and this observation was in line with their published potencies at the murine Gpr119 receptor (Overton et al., 2006). Using apical PSN632408 (10 μM) as the stimulus of choice, we then established that the reductions in I_sc_ to Gpr119 activation were unaltered by pretreatment with the DPP-IV inhibitor (compound 3) (Lankas et al., 2005), which has been shown to selectively amplify Y_2_ receptor- but not Y_1_-mediated antisecretory effects (Cox, 2008). Colonic PSN632408 responses were, however, inhibited significantly by the Y_1_ receptor antagonist (BIBO3304; as were OEA responses) (Figure S3B), but they were not affected by Y_2_ antagonism with BIIE0246 (Figure 2B). Thus, Gpr119 responses in WT colon are Y_1_ receptor mediated and are insensitive to DPP-IV blockade.

Next, we set out to establish whether neural NPY was necessary for Gpr119 activity. To do this, we first investigated whether WT PSN632408 responses were sensitive to pretreatment with tetrodotoxin (TTX, 100 nM) and found that they were not (−12.4 ± 2.9 μA/cm^2^, n = 4 compared with controls in Figure 2B). We then compared mucosal responses to PSN632408, PSN375963, or OEA (at 10 μM) in WT colon with those from *NPY*^−/−^, *PYY*^−/−^, and *NPYPYY*^−/−^ colon (Figures 2C, S3B, and S3C). Apical responses to the three agonists were unchanged (compared to WT responses) in *NPY*^−/−^ mucosae, but all three were significantly inhibited in colon from *PYY*^−/−^ and *NPYPYY*^−/−^ mice. The residual apical PSN632408 responses in the latter two null tissues were the same as vehicle controls (2.0 ± 1.4 μA/cm^2^, dashed lines). Apical OEA responses were also not significantly different in *NPY*^−/−^ compared to WT colon, and they were abolished by Y_1_ antagonism in tissue from both genotypes (Figures S3B and S3C). Basolateral PSN632408 responses were also inhibited significantly in *PYY*^−/−^ (2.0 ± 0.9 μA/cm^2^, n = 4) and *NPYPYY*^−/−^ tissue (2.0 ± 0.8 μA/cm^2^, n = 4, with vehicle controls of 3.0 ± 3.3 μA/cm^2^, n = 4) compared with WT basolateral PSN632408 responses (−11.9 ± 3.5 μA/cm^2^, n = 4). This demonstrates that ablation of PYY or selective blockade of epithelial Y_1_ receptors (Figure 2B) renders colonic mucosa insensitive to Gpr119 activation, confirming the functional requirement of Gpr119 mucosal signaling for endogenous PYY.

Taken together, these findings indicate that apical or basolateral activation of Gpr119 results in a PYY-mediated, Y_1_ receptor-specific epithelial response that is observed along the length of the intestine and is greatest in the descending colon. Mucosal responses to Gpr119 agonists do not involve enteric nerves (or NPY) and are not amplified by blockade of DPP-IV activity.

### Gpr119 Agonist Responses in Human Colon Mucosa

Since Gpr119 expression is also significant in human colon (Chu et al., 2008), we set out to establish whether mucosal Gpr119 activities were similar in normal human colonic mucosa. Apical PSN632408 (10 μM) (Figure 2D) responses closely resembled those of WT mouse colon (Figure 1B), although basolateral responses in human colon mucosa were slower in onset, probably due to the barrier effect of basolateral connective tissue.

Also consistent with our mouse tissue studies, Gpr119 responses in human colon were only blocked by the Y_1_ receptor antagonist, BIBO3304 (Figure 2E). Gpr119 responses in human colon were unaffected by pretreatment with the GLP-1 antagonist exendin(9-39) alone (Figure 2E), which abolished exendin 4 (100 nM) responses (6.2 ± 2.2 μA/cm^2^ versus −1.9 ± 0.5 μA/cm^2^, n = 4, p < 0.05). These results are consistent with our finding that DPP-IV inhibition, which prolongs GLP-1 action, did not affect the mucosal Gpr119 responses in mouse colon. Furthermore, neither Y_2_ antagonism with BIIE0246 nor DPP-IV inhibition had any effect on Gpr119 responses in human colon (Figure 2E), again, as observed in WT mouse colon. However, in the presence of both Y_1_ and Y_2_ antagonists, a small but significant increase in I_sc_ was observed following apical PSN632408 addition to human colon. This response was abolished by the GLP-1 receptor antagonist exendin(9-39) (Figure 2E), indicating that corelease of endogenous GLP-1 with PYY occurs in human colon mucosa following Gpr119 stimulation. Only when the Y_1_ and Y_2_ receptors were blocked was the small GLP-1 secretory (presumably G_αs_-coupled) signal revealed in human tissue. As seen in mouse mucosae, PSN375963 and OEA (at 10 μM) also reduced I_sc_ to levels similar to those observed with PSN632408 in human colon (Figure 2F). Additionally, PSN632408 responses were unaffected by nerve block with TTX, resulting in reductions in I_sc_ (−10.4 ± 1.4 μA/cm^2^, n = 3) that were not significantly different from controls (Figure 2F).

Taken together, these data suggest that in human colon, PYY mediates the predominant antisecretory effects following Gpr119 stimulation and that this mechanism is epithelial in origin and exclusively Y_1_ receptor mediated. Endogenous GLP-1 (and GLP-2) meanwhile exert minor exendin(9-39)-sensitive electrogenic responses that are only observed when Y_1_ and Y_2_ receptors are blocked.

### Endogenous GLP-1 Does Not Mediate Gpr119 Responses, and Plasma GLP-1 Levels Are Unaltered in Null Mice

**Figure 3:**
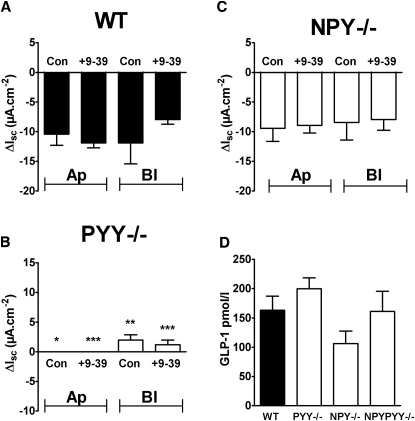
Murine Gpr119 Responses Are Not Sided or Dependent on GLP-1, and Plasma GLP-1 Levels Are Similar across the Genotypes (A–C) PSN632408 (10 μM) responses after apical (Ap) or basolateral (Bl) addition ± pretreatment with exendin(9-39) (1 μM) in WT colon (n = 4) (A), in *PYY*^−/−^ colon (n = 5) (B), or in *NPY*^−/−^ colon mucosae (n = 4) (C). Significant differences between *PYY*^−/−^ and WT PSN632408 responses are shown (^∗^p ≤ 0.05, ^∗∗^p ≤ 0.01, ^∗∗∗^p ≤ 0.001), and values are the mean ±SEM throughout. (D) Plasma levels of GLP-1 in each knockout are not significantly different from WT levels. Values are the mean + SEM (n = 3).

In WT mouse colon mucosa, neither apical nor basolateral PSN632408 responses were significantly affected by the GLP-1 receptor antagonist exendin(9-39) (Figure 3A). Gpr119 responses were absent from *PYY*^−/−^ colon, and here too exendin(9-39) had no effect on either apical or the residual basolateral responses (Figure 3B). Notably, Gpr119 responses were unaltered in *NPY*^−/−^ colon, and again these mucosal responses were insensitive to exendin(9-39) (Figure 3C). Thus, the Gpr119 mucosal responses in the mouse colon are not dependent on GLP-1. In addition, we observed small apical PSN632408 responses (−3.0 ± 1.7 μA/cm^2^, n = 4) in WT jejunum mucosa that were absent in *PYY*^−/−^ jejunum (0.0 ± 0.0 μA/cm^2^, n = 4).

To confirm that exendin(9-39) (1 μM) blocked mucosal GLP-1 receptors, we first monitored (in WT mouse colon) small increases in I_sc_ to basolateral addition of the GLP-1 agonist exendin 4 (100 nM, 4.4 ± 1.2 μA/cm^2^, n = 10). Pretreatment with exendin(9-39) (1 μM) inhibited these responses (0.8 ± 0.8 μA/cm^2^, n = 5, p = 0.07) and significantly reduced exendin 4 responses in *PYY*^−/−^ colon (controls, 6.6 ± 1.3 μA/cm^2^; after exendin(9-39), 0.7 ± 0.7 μA/cm^2^; both n = 3, p ≤ 0.01). Thus, a small secretory GLP-1 response was revealed using exendin 4 in WT colon mucosa, and this was unchanged in *PYY*^−/−^ tissue.

Therefore, Gpr119 mucosal sensitivity depends primarily on PYY in both the small and large bowel of the mouse, with endogenous GLP-1 playing no significant acute role in the colonic Gpr119 response. It should also be noted that the plasma GLP-1 levels of null mice were not significantly different from those of their WT counterparts (Figure 3D).

### Gpr119 Responses Are Glucose Sensitive in Mouse and Human Colon Mucosae

**Figure 4:**
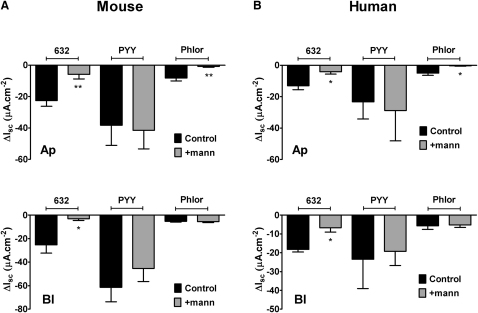
Mouse and Human Colon Apical and Basolateral PSN632408 Responses Are Glucose Sensitive (A) Glucose sensitivity of apical (Ap, n = 7 throughout) or basolateral (Bl, n = 6 throughout) PSN632408 (10 μM) responses in mouse colon mucosa in the presence of glucose on both sides (11.1 mM, Control) or following mannitol replacement (11.1 mM, + mann) on either side. PYY (10 nM, added basolaterally only) and phloridzin (Phlor, 50 μM, apical only) responses are also shown. Statistical comparisons are made between agonist or phloridzin responses obtained from tissue bathed with glucose on both sides (Control) and single-sided mannitol replacement. ^∗^p ≤ 0.05, ^∗∗^p ≤ 0.01. (B) Apical (Ap, n = 3) and basolateral (Bl, n = 3) responses to PSN632408 (10 μM) in human colon mucosa, either in the presence of glucose (black bars) or after mannitol (11.1 mM, + mann, gray bars) replacement on either side. Values are the mean − SEM throughout. ^∗^p ≤ 0.05.

Glucose modulates the activity of isolated L cells (Reimann et al., 2008). In order to establish the glucose-sensitivity of L cells in intact tissue, the response to Gpr119 activation was analyzed in mucosal preparations from mouse and human colon. When glucose was replaced with mannitol in either reservoir (coincident with the side of PSN632408 addition), the Gpr119 responses were significantly reduced in mouse (Figure 4A) and human colon mucosa (Figure 4B). In contrast, PYY responses per se were not glucose sensitive, nor were the antisecretory effects of the α_2_-adrenoceptor agonist, UK14,304 (data not shown). The sodium/glucose cotransport inhibitor, phloridzin (added apically throughout), was only effective when glucose was present apically in both preparations (Figures 4A and 4B, upper graphs). Basolateral mannitol replacement predictably had no effect on the small phloridzin reductions in I_sc_ (Figures 4A and 4B, lower plots) because the glucose transporter SGLT1 is targeted apically. Thus, apical and basolateral Gpr119 receptors can be activated by PSN632408 in a glucose-sensitive manner to cause consequent reductions in I_sc_ from mouse and human colonic L cells.

To test whether K_ATP_ channels, known to be involved in glucose sensing in isolated L cells (Reimann et al., 2008), were involved in basal or Gpr119-activated responses, the K_ATP_ channel blocker tolbutamide was used (at 1 mM throughout) in the presence of glucose. First, apical tolbutamide per se reduced I_sc_ by −17.1 ± 1.5 μA/cm^2^ (n = 5) in WT tissue, and the blocker was significantly less effective in *PYY*^−/−^ colon (−5.3 ± 1.3 μA/cm^2^, n = 3, p ≤ 0.01) and in Y_1_ antagonist-pretreated WT tissue (−8.9 ± 2.4 μA/cm^2^, n = 4, p ≤ 0.05 compared with controls). Basolateral tolbutamide exerted an effect similar to apical addition in WT tissue, but the reductions in I_sc_ were more variable (data not shown). PYY was released from WT colonic mucosa treated with tolbutamide, but not from vehicle controls, and WT tissue total PYY levels were not altered significantly (Figure S3D). In WT colon mucosa exposed to apical tolbutamide, subsequent apical PSN632408 responses were reduced (from −10.9 ± 1.7 μA/cm^2^, n = 6 to −6.6 ± 1.3 μA/cm^2^, n = 3), but this did not reach statistical significance. We conclude that blockade of K_ATP_ channels is more efficacious in WT than in *PYY*^−/−^ mucosa, and we suggest that the larger tolbutamide responses observed in WT tissue could be a consequence of enhanced PYY release and Y_1_ receptor activation. In the presence of glucose and endogenous PYY, apical Gpr119 activation was partially inhibited by K_ATP_ channel blockade.

### PYY Mediates the Gpr119 Agonist-Induced Improvement in Glucose Tolerance

**Figure 5:**
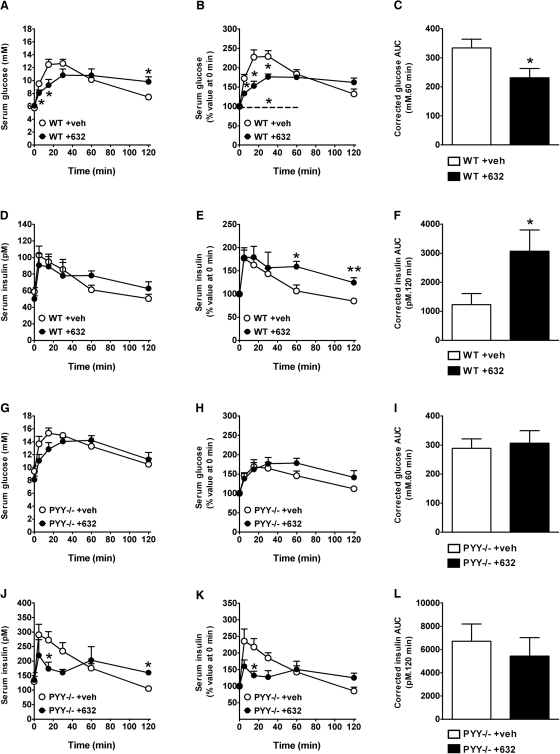
Oral PSN632408 Improves Oral Glucose Tolerance in WT but Not *PYY*^−/−^ Mice (A–L) Absolute levels of serum glucose (A and G) and percent of serum glucose values at time 0 min (B and H) are shown with the areas under the resultant glucose curves (0–60 min) (C and I). The dashed line (B) denotes a significant difference between vehicle and treated groups during 0–60 min by repeated measures. Serum insulin levels (D and J) are also expressed as a percent of values at time 0 (E and K), with the areas under the resultant insulin curves (0–120 min) (F and L) in 24 hr fasted WT (A–F) and *PYY*^−/−^ (G–L) mice after voluntary oral consumption of glucose (3 g/kg body weight). At 30 min prior to glucose consumption, mice had voluntarily consumed a paste containing PSN632408 (+632) at a dose of 100 mg/kg or a vehicle control paste (+veh). Data are means + SEM from 7–8 mice per group. ^∗^p ≤ 0.05 and ^∗∗^p ≤ 0.01 versus vehicle-treated control mice of the same genotype at specific times.

As Gpr119 agonism with PSN632408 induces effects on intestinal function via PYY, we sought to determine whether PYY mediated other physiological functions of Gpr119 agonism, such as glucose tolerance. The Gpr119 agonist AR231453 has been shown previously to suppress glycemic excursions after oral or intraperitoneal (i.p.) glucose administration, particularly when glucose was administered orally (Chu et al., 2007). We chose the oral route of administration for both PSN632408 and glucose to maximize effects via the intestine (Chu et al., 2007). Our data show that Gpr119 agonism with orally administered PSN632408 significantly reduced glycemic excursions after oral glucose ingestion in WT mice (Figures 5A and 5B) and resulted in a significant decrease in the area under the curve during the first 60 min after glucose ingestion (Figure 5C). This effect of PSN632408 on glucose tolerance was associated with a significantly greater plasma insulin response to oral glucose, indicated by a longer-lasting elevation of serum insulin concentrations after glucose ingestion and a significant increase in the area under the resultant curve (Figures 5D, 5E, and 5F, respectively). Interestingly, these PSN632408 effects were abolished in *PYY*^−/−^ mice (Figures 5G–5L), demonstrating that PYY is required for Gpr119-induced improvement in glucose tolerance and stimulation of circulating insulin levels.

## Discussion

**Figure 6:**
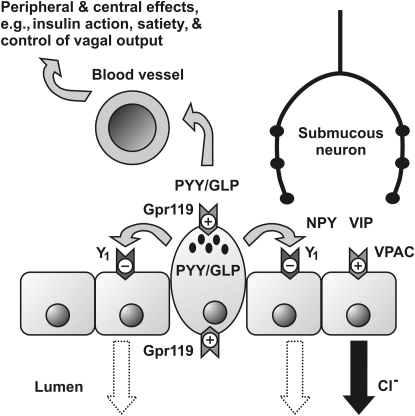
The Proposed Paracrine Effects of Gpr119 Activation in Human and Mouse Colon Mucosa Stimulation of L cell Gpr119 (a G_αs_-coupled mechanism) results in degranulation and release of PYY and GLPs (GLP-1 and GLP-2). PYY inhibits epithelial ion secretion via basolateral Y_1_ receptor (G_αi_-coupled) inhibition of cAMP-dependent Cl^−^ secretion, a process initially activated, for example, via VIP released from intramural submucosal neurons, resulting in VPAC receptor (G_αs_-coupled) activation to increase intraepithelial cAMP and Cl^−^ secretion (solid arrow). PYY-mediated inhibition of this process (denoted by dotted arrows in lumen) results following epithelial Y_1_ receptor activation. GLP-1 has limited G_αs_-coupled activity in human epithelia. Additionally, PYY and GLPs enter the bloodstream, exerting their hormonal influences on peripheral and central targets, i.e., regulating insulin action, inducing satiety, and controlling vagal activity.

Our study demonstrates that glucose-sensitive Gpr119 activation causes electrolyte antisecretory effects that are most likely due to an increase in endogenous PYY release subsequently activating epithelial Y_1_ receptors (Figure 6) together with improved oral glucose tolerance. Importantly, the antisecretory effects following Gpr119 activation are absent from *PYY*^−/−^ and *NPYPYY*^−/−^ but not from *NPY*^−/−^ mouse GI tissues, consistent with mediation of this process by PYY rather than NPY. Our results also demonstrate that L cell-derived PYY and GLP-1 differentially mediate Gpr119 mucosal responses in human colon and that in mouse colon, the Gpr119-induced, PYY-mediated antisecretory response is partially dependent on K_ATP_ channel activity. This L cell mechanism is similar to that described recently for GLP-1 release from isolated L cells (Lauffer et al., 2009; Tolhurst et al., 2009), with the notable difference being that Gpr119 receptors are present within both apical and basolateral membranes of intact tissue where epithelial polarity is maintained. In addition to PYY's ability to slow gastric emptying and regulate satiety and glucose homeostasis, it also mediates mucosal responses to Gpr119 stimulation in the small intestine, where efficacy is predictably reduced, correlating with the lower L cell frequency in this region relative to the distal bowel (Arantes and Nogueira, 1997; Sundler et al., 1993). Thus, we conclude that intramural and lumenal fatty acid amides have the potential to activate L cells from either a blood-borne or lumenal direction to cause PYY/GLP-1 corelease (Figure 6), and this can occur along the length of the GI tract. While local GLP-1 and PYY activities differ, e.g., the former modulating epithelial barrier function rather than modulating epithelial anion secretion, the repertoire of GLP-1 and PYY hormonal activities match more closely, e.g., both reduce gastric emptying, inhibit intestinal motility, and modulate vagal afferent output (Drucker, 2005; Dockray, 2009). Mucosal Gpr119 responses were not amplified by DPP-IV inhibition, indicating that degradation of full-length PYY to PYY(3-36) is not significant following Gpr119 activation in human or mouse colon mucosa. We therefore conclude that antidiabetic DPP-IV inhibitors such as vildagliptin may cause constipation, as has been observed clinically (Lauster et al., 2007), but that this is unlikely to involve increased stability of PYY.

Results from our mechanistic investigations corroborate recent studies utilizing purified and single-mouse L cells (Reimann et al., 2008) and signaling studies utilizing endocrine L cell-containing lines (Chu et al., 2008; Lauffer et al., 2009). However, our study also demonstrates that PYY is required for the effect of oral Gpr119 agonism with PSN632408 to improve oral glucose tolerance and stimulate circulating insulin levels. As Gpr119 is expressed in L cells that contain GLP-1, it has been hypothesized that Gpr119 agonism may improve glucose tolerance via stimulation of GLP-1 release, as recently reviewed (Overton et al., 2008). However, PYY is copackaged with GLPs in L cells (Böttcher et al., 1984) and has been shown to stimulate insulin sensitivity and improve glucose disposal after acute (van den Hoek et al., 2004) or chronic (Pittner et al., 2004; van den Hoek et al., 2007) administration to rodents. Our finding that oral PSN632408 improved oral glucose tolerance and enhanced glucose-induced increases in circulating insulin levels in WT but not in *PYY*^−/−^ mice is consistent with the possibility that orally administered PSN632408 can improve oral glucose tolerance by L cell Gpr119 agonism with subsequent PYY-mediated effects.

Taken together, these data suggest that Gpr119 has significant effects upon intestinal mucosal function, as well as other physiological outcomes such as glucose tolerance, and PYY is critical for these effects. Establishing the full repertoire of Gpr119-activated intestinal mechanisms that enhance not only GLP-1 but also PYY-mediated responses with consequent antihyperglycemic effects now provides an optimal platform for a high-affinity Gpr119 agonist to treat diabetes and obesity.

## Experimental Procedures

### Targeted Deletion of PYY and NPY

*PYY*^−/−^ and *NPY*^−/−^ mice were generated by homologous recombination in embryonic stem cells, as described previously (Boey et al., 2006; Karl et al., 2008). *NPY*^−/−^ and *PYY*^−/−^ mice were crossed to generate double heterozygotes and subsequent double knockout *NPYPYY*^−/−^ mice, which was confirmed by Southern blot analysis and immunohistochemistry (Doyle et al., 2008). All mice were on the same C57BL/6-129/SvJ background and had free access to standard chow and water ad libitum. Where possible, WT littermates were used as controls. Importantly, there was no difference between the WT littermates derived from heterozygous breeding compared to WT mice bred separately out of these lines, in any of the parameters investigated here or in others not shown here. Mice were killed by a Schedule 1 method and tissues were excised for in vitro experimentation.

### Immunohistochemistry

Lengths (2–3 cm) of mouse descending colon were washed in Krebs-Henseleit (KH) buffer, immersed in PFA (4%) for a minimum of 24 hr, washed in PBS, and cryoprotected in 30% sucrose in PBS for 48 hr before embedding in optimal cutting temperature (OCT) compound and storage at −80°C. Sections (15 μm) were cut, rehydrated in PBS, and blocked in 10% normal goat serum in PBS for 2 hr before incubating overnight in either polyclonal anti-PYY antibody (1:1000) to visualize PYY-containing endocrine cells or in chromogranin A (1:400) to label all endocrine cells. Longer incubation times (3–4 days) were used to enable anti-NPY labeling (1:400) or protein gene product (PGP) 9.5 (1:400) labeling of all enteric neurons. Primary antibodies were visualized with goat anti-rabbit F(ab')_2_ secondary antibodies conjugated to either FITC or TRITC (1:200, 2 hr at room temperature), and nuclei were visualized with DAPI (1:1000 in PBS for 2 min). Sections were washed in PBS, mounted in FluorSave, and viewed with a Provis microscope fitted with appropriate filters and AxioVision software. The numbers of endocrine cells were counted per section by an unbiased observer.

### Electrophysiology

Colonic mucosa from clinical specimens or from WT or knockout male mice (>15 weeks old) was voltage clamped at 0 mV in Ussing chambers, as described previously (Cox and Tough, 2002; Cox et al., 2001). Vectorial ion transport was measured continuously as I_sc_ (μA/cm^2^), and all peptide additions were basolateral, as receptors are targeted to the basolateral epithelial domains. Gpr119 agonist (PSN632408, PSN375963, or OEA) additions were made to either the apical or basolateral reservoirs 15–20 min following VIP (10 nM). This is approximately the EC_50_ concentration of VIP in mouse mucosa (Cox et al., 2001) and an optimal secretory pretreatment for revealing subsequent G_αi_-coupled epithelial responses in mouse mucosae.

Once stable I_sc_ levels were achieved, mucosae were treated with the DPP-IV inhibitor (1 μM compound 3) (Lankas et al., 2005), neuronal activity was abolished with TTX (100 nM), or endogenous GLP-1 responses were inhibited with exendin(9-39) (1 μM). Treatment periods were 20–30 min prior to addition of the Y_1_ receptor antagonist BIBO3304 (BIBO; 300 nM) or the Y_2_ selective antagonist BIIE0246 (BIIE; 1 μM). A concentration of 10 μM PSN632408 was chosen as the Gpr119 stimulus, as it resulted in near maximal responses in mouse colon mucosa. Control experiments with Y agonists utilized concentrations that preferentially stimulated either Y_1_ receptors (10 nM Pro^34^PYY), Y_2_ receptors (30 nM PYY(3-36)), or Y_4_ receptors (30 nM rPP), as optimized in previous studies (Cox et al., 2001; Tough et al., 2006). Y agonist-induced reductions in I_sc_ in epithelia are a result of G_αi_-coupled attenuation of cAMP levels with consequent long-lasting decreases in Cl^−^ ion secretion (Cox et al., 1988). For TRPV1 desensitization, two 1 μM additions of capsaicin (to both sides) were made, followed by VIP (10 nM) and then either apical PSN632408 or OEA (10 μM) at 10 min intervals.

In glucose sensitivity studies, tissues were bathed with KH buffer containing glucose (11.1 mM) on one side and mannitol (11.1 mM) in place of glucose on the other. Tolbutamide (1 mM) was used to block apical K_ATP_ channels, and changes in basal I_sc_ levels and subsequent Gpr119 responses were recorded.

### Peptide Levels and PYY Release

Plasma GLP-1 was measured in duplicate by an established in-house radioimmunoassay (Kreymann et al., 1987). The antibody cross-reacted 100% with all amidated forms of GLP-1 but did not cross-react with glycine-extended forms (GLP-1(1-37) and GLP-1(7-37)) or any other GI peptides.

For PYY release, mucosae were incubated in 2 ml KH buffer at 37°C with either vehicle (1% DMSO) or tolbutamide (1 mM) for 90 min. PYY-like immunoreactivity was measured by an established radioimmunoassay (Adrian et al., 1985) using antiserum Y21 (at a final dilution of 1:50,000) that cross-reacted with all biologically active forms of PYY, but not NPY, PP, or other peptides. The assay was performed in 0.7 ml KH containing 0.3% BSA and was incubated for 3 days at 4°C before separation of free and antibody-bound label.

### Oral Glucose Tolerance Test

Male WT and *PYY*^−/−^ mice at 19–24 weeks of age were used. The oral route of administration of both PSN632408 and glucose was chosen because GI effects contribute to effects of Gpr119 agonism. Notably, the effectiveness of the Gpr119 agonist AR231453 to improve glucose tolerance was reduced by almost 50% when glucose was given i.p. compared to oral delivery, suggesting incretin involvement in this effect (Chu et al., 2007). Because stress is known to mask physiological responses to gut hormones (Abbott et al., 2006), we trained mice to voluntarily eat a vehicle paste followed 30 min later by a vehicle jelly that would contain PSN632408 and glucose, respectively, on the day of experimentation, thereby avoiding the stress of oral drug and glucose administration by gavage. Training and vehicle jelly preparation were as described previously (Zhang et al., 2010).

Mice were fasted for 24 hr and were then given either vehicle paste or paste containing 13.5 mg/ml PSN632408 (100 mg/kg) in 24% Gelucire 44/14 and 76% aqueous solution. PSN632408 (25 mg) was first suspended at 65°C in 450 μl preheated Gelucire 44/14. Aqueous solution (1.4 ml) containing 22.1% wt/vol Splenda low calorie sweetener and 7.1% vol/vol imitation strawberry flavoring essence was then added to the Gelucire 44/14 and mixed to form a paste. At 30 min after mice had consumed the entire PSN632408 or vehicle paste, an oral glucose bolus (3 g/kg) was delivered as a glucose jelly. To this end, glucose (0.52 g/ml) was incorporated into a jelly containing 4.9% wt/vol gelatin and 7.5% imitation strawberry flavoring essence. Tail vein blood was collected at 0, 5, 15, 30, 60, and 120 min after the mouse had finished eating the glucose jelly, and serum was produced for the determination of glucose and insulin levels using a glucose oxidase assay and ELISA, respectively.

Glucose tolerance curves for serum glucose and insulin are presented as absolute values, as well as percent serum glucose or insulin concentrations prior to glucose ingestion. Additionally, absolute areas under the serum glucose or insulin concentration curves were calculated (after subtracting glucose or insulin concentrations prior to glucose ingestion) between 0 and 60 min (for glucose) or 0 and 120 min (for insulin) after glucose ingestion and are referred to as area under the curve.

### Data Analysis

Functional data from GI tissues measuring the maximal changes in I_sc_ are expressed as the mean ±SEM per unit area (cm^2^). Single comparisons were performed using Student's unpaired t test, whereas multiple comparisons utilized one-way ANOVA with Dunnett's post hoc test. Changes in PYY release were compared using Student's paired t test, and in all cases p ≤ 0.05 was considered significantly different. Data from in vivo analyses are expressed as means ±SEM. Differences among groups of mice were assessed by repeated-measures ANOVA with genotype and treatment as main effects (Statistical Package for the Social Sciences, SPSS Inc., version 17.0). Statistical significance was defined as p ≤ 0.05.

### Materials

BIBO3304 and BIIE0246 were gifts from Boehringer-Ingelheim Pharma KG (Biberach an der Riss, Germany), and stock solutions were dissolved in 10% DMSO (at 1 mM) and stored at −20°C until required. All peptides were from Bachem Laboratories (St. Helens, UK). Stocks were dissolved in water, and aliquots were stored at −20°C, undergoing a single freeze-thaw cycle. The DPP-IV inhibitor compound 3 was from R. Roy (Merck, Rahway, NJ) (Lankas et al., 2005). PSN632408, PSN375963, and OEA were purchased from Cayman Chemical (Ann Arbor, MI) and GW6471 from Tocris Bioscience (Bristol, UK). Anti-PYY (from E. Ekblad, University of Lund, Sweden), anti-NPY antibodies (Affiniti Research Products Limited, Exeter, UK), goat anti-rabbit FITC- or TRITC-conjugated secondary antibodies (Chemicon, Harrow, UK), DAPI (Sigma-Aldrich, Poole, UK), PGP9.5 (Ultraclone Ltd., Isle of Wight, UK), and chromogranin A (DAKO A/S, Glostrup, Denmark) were reconstituted and stored as recommended by each supplier. OCT and polysine-coated slides were from VWR International (Lutterworth, UK) and FluorSave from Calbiochem (Nottingham, UK). Materials used for in vivo experimentation were: Splenda Low-Calorie Sweetener (Johnson & Johnson Pacific Pty Ltd, Ultimo, Australia), Gelucire 44/14 (Gattefossé, Saint Priest, France, a gift from J. Pinder, Trapeze Associates Pty Ltd, Bella Vista, New South Wales, Australia), imitation strawberry flavoring essence (Queen Fine Foods Pty Ltd, Alderley, Queensland, Australia), glucose (Sigma, St. Louis), gelatin (Gelita Australia Pty Ltd, Botany, New South Wales, Australia), glucose oxidase assay kit (Trace Scientific, Noble Park, Victoria, Australia), and insulin ELISA kit (Crystal Chem, Downers Grove, IL). All other compounds were of analytical grade from Sigma-Aldrich (Poole, UK).

## Supplemental Information

Document S1. Three Figures and One Table
