# Supplementary material for: Peptide YY Is Critical for Acylethanolamine Receptor Gpr119-Induced Activation of Gastrointestinal Mucosal Responses
Source: Cell Metab. 2010 Jun 9;11(6):532–42. doi: 10.1016/j.cmet.2010.04.014 (PMC2890049; doi:10.1016/j.cmet.2010.04.014)
Supplement: Document S1. Three Figures and One Table [file mmc1.pdf]

Supplemental Information
Cell Metabolism, *Volume 11*

Peptide YY Is Critical for Acylethanolamine Receptor Gpr119-Induced Activation of Gastrointestinal Mucosal Responses

Helen M. Cox, Iain R. Tough, Anne-Marie Woolston, Lei Zhang, Amy D. Nguyen, Amanda Sainsbury, and Herbert Herzog

Table S1. Ages, Gender, Weights and Baseline Electrophysiological Characteristics in Colon Mucosae from Human and Mouse

	Age	Gender	Weight (g)	Resistance ($\Omega.cm^2$)	Basal I_{sc} ($\mu A.cm^{-2}$)
Human colon	66.0 ± 4.2 yr (11)	8M 3F	-	72.4 ± 3.5 (68)	87.1 ± 6.6 (68)
Mouse colon					
WT	18.8 ± 0.7 wk (32)	30M 2F	30.8 ± 0.7 (32)	35.1 ± 1.2 (151)	59.7 ± 3.5 (151)
PYY-/-	19.1 ± 2.2 wk (16)	15M 1F	34.6 ± 0.8 (16)*	30.5 ± 1.9 (61)	70.8 ± 5.0 (61)
NPY-/-	21.6 ± 1.4 wk (14)	10M 4F	30.0 ± 1.2 (14)	30.5 ± 1.5 (49)	49.4 ± 5.5 (49)
NPYPYY-/-	16.2 ± 1.0 wk (11)	7M 4F	31.3 ± 2.1 (11)	30.8 ± 2.1 (24)	53.3 ± 7.6 (24)

Related to Figure 1. Each value is the mean ± SEM from the number of observations (in parenthesis). Statistical comparisons showed one significant difference; * $P \leq 0.05$, between $PYY^{-/-}$ and WT mouse weights.

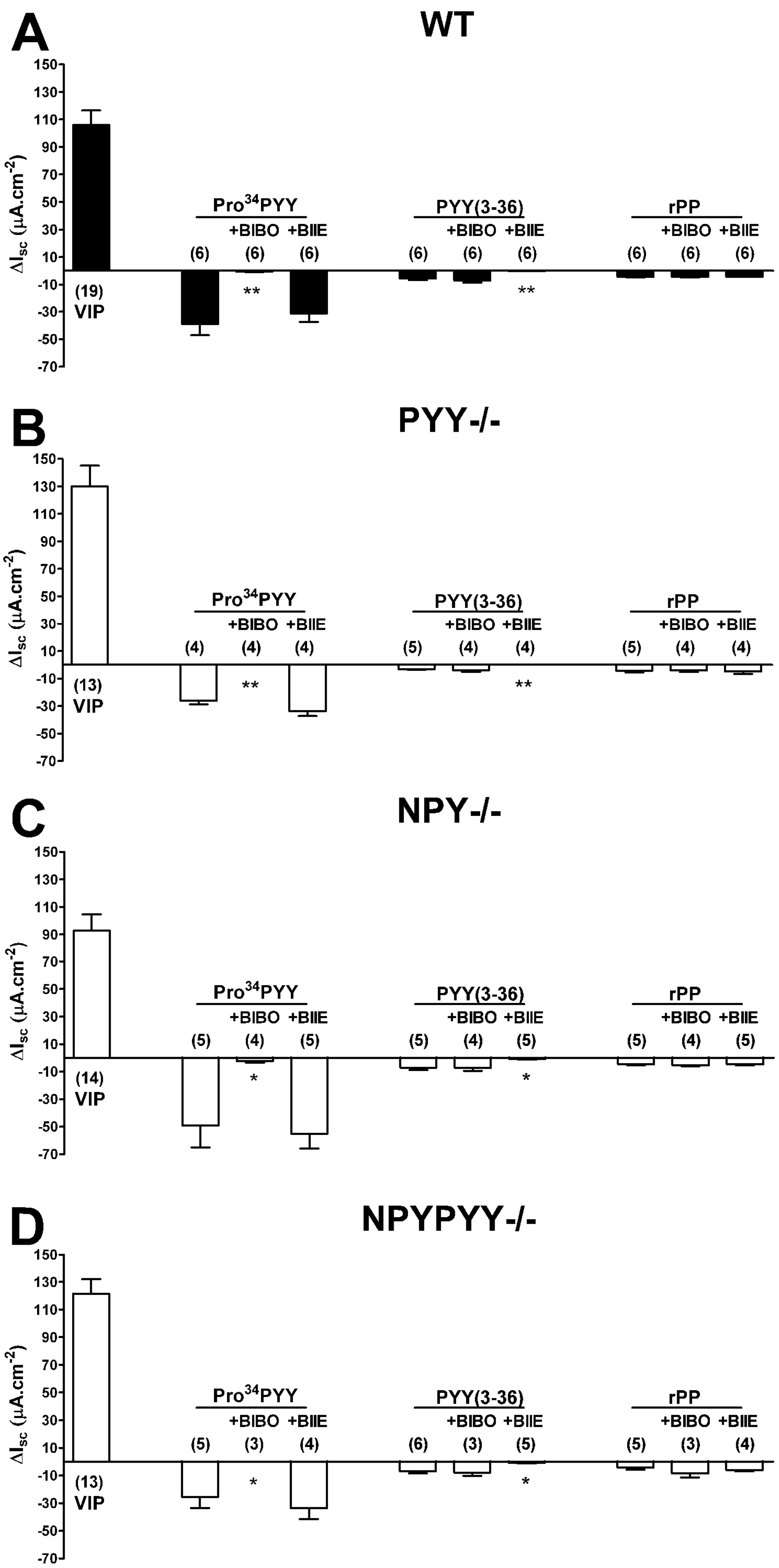
A
WT
B
PYY-/-
C
NPY-/-
D
NPYPYY-/-
ΔIsc (μA.cm-2)
150
130
110
90
70
50
30
10
-10
-30
-50
-70
Pro34PYY
PYY(3-36)
rPP
+BIBO
+BIIE
VIP
(19)
(13)
(14)
(13)
(6)
(5)
(4)
(3)
**
*

Figure S1, related to Figure 1.

(A-D) Responses to different Y agonists following VIP (30 nM) pretreatment. Subsequent decreases in I_{sc} were measured after addition of either Pro^{34}PYY (10 nM), PYY(3-36) (30 nM) or rPP (30 nM) in the absence or presence of either BIBO3304 (300 nM, Y_1 antagonism) or BIIE0246 (1 µM, Y_2 antagonism). Each bar is the mean ± SEM from the numbers of observations shown in parenthesis. Significant differences between control agonist and antagonist-pretreated responses are shown by * $P \leq 0.05$ and ** $P \leq 0.01$.

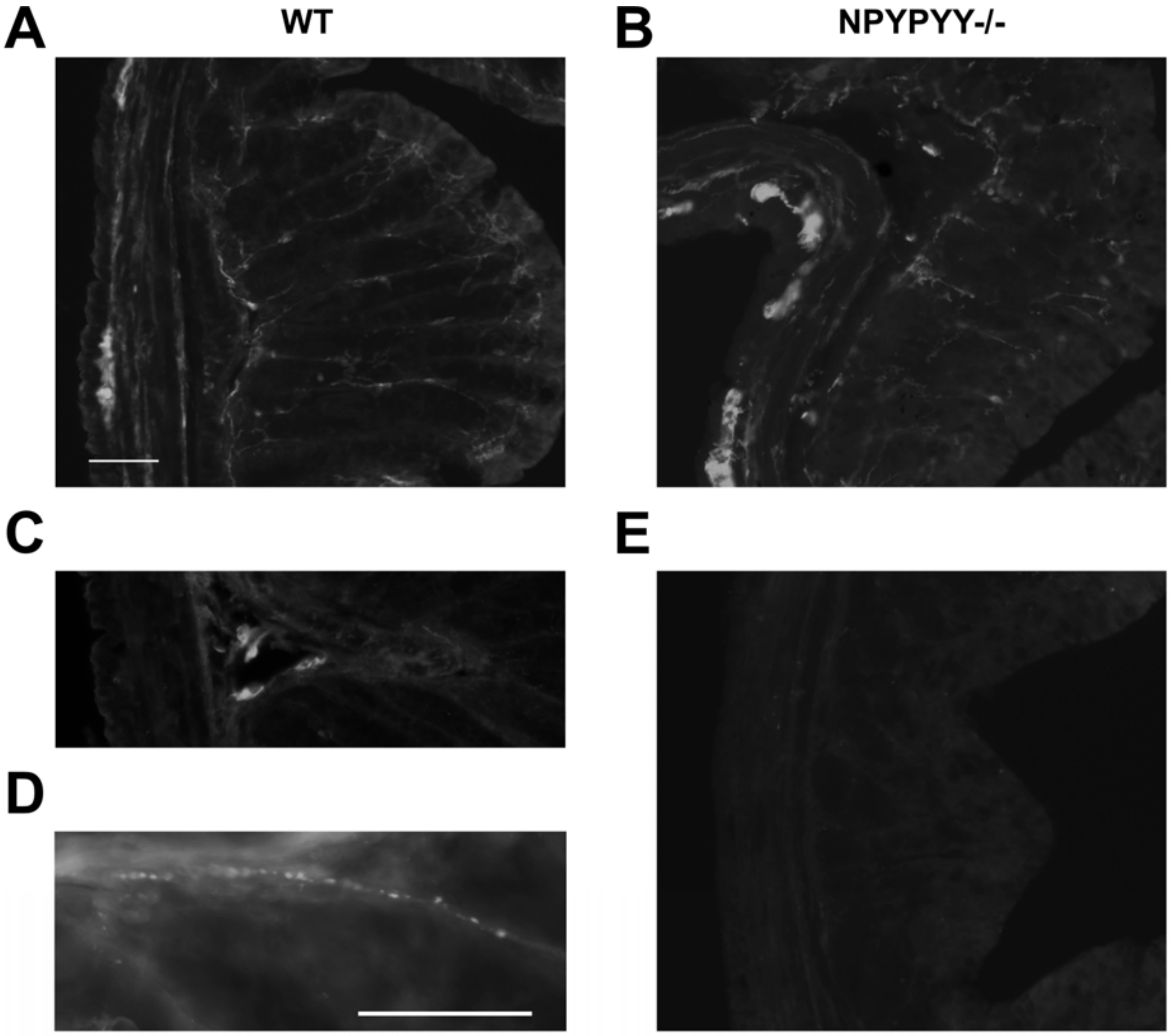

Figure S2, related to Figure 1.

Innervation of colon mucosa from WT (A) and *NPYPYY*$^{-/-}$ (B) mice. Extensive and similar PGP9.5 labelling is seen within myenteric and submucous ganglia as well as circular smooth muscle and submucosal regions of tissue from both genotypes. The bar in A = 20 µm and this scale applies to B and E. NPY-immunolabelling in WT colon showing several cell bodies in a submucous plexus (C) and a varicose fibre (D, bar = 50 µm and applies to C) compared with the absence of any NPY-labelling in *NPYPYY*$^{-/-}$ tissue (E).

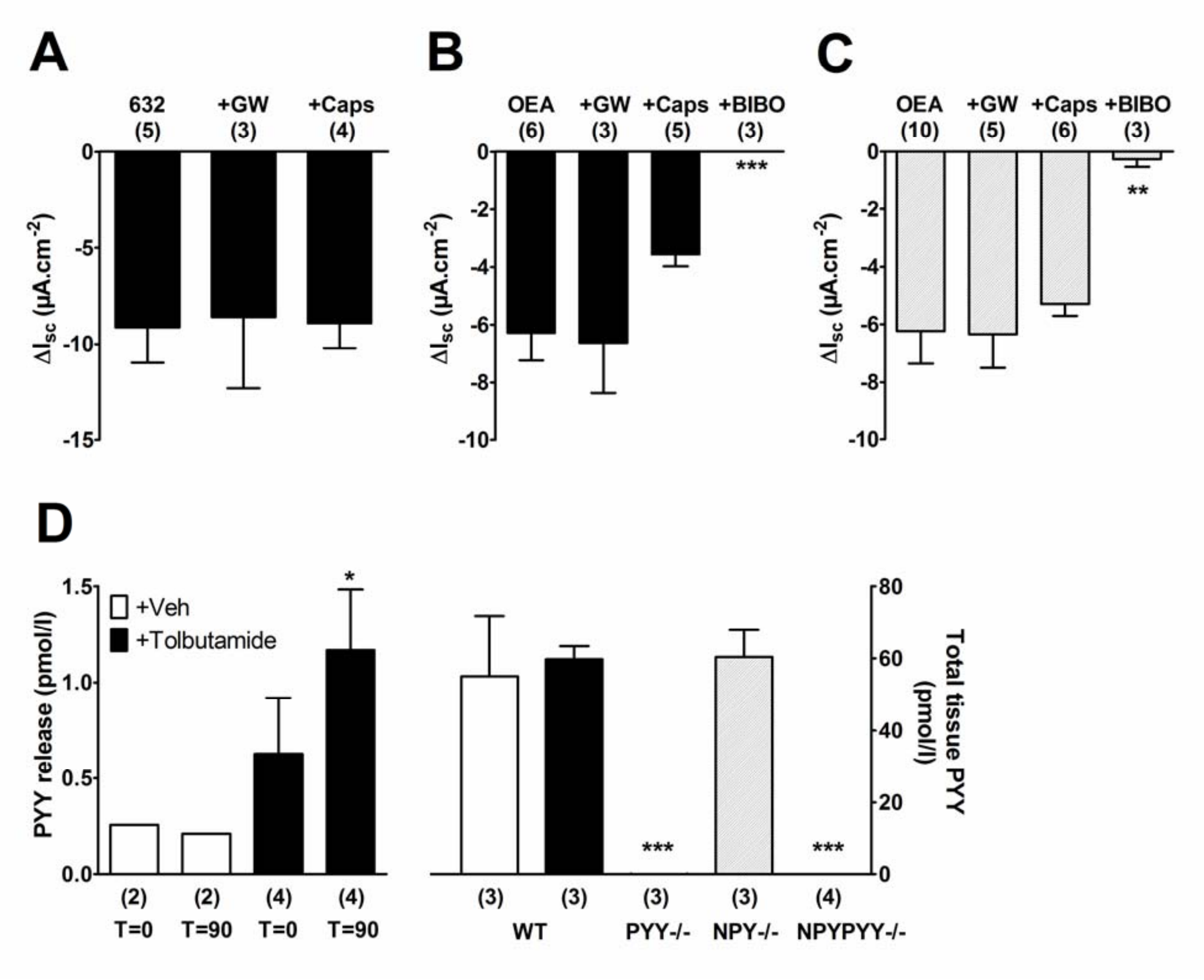

Figure S3, related to Figure 2.

Sensitivity of WT (A and B) and *NPY*$^{-/-}$ (C) colon mucosa to apical PSN632408 or OEA (both at 10 μM) or following GW6471 (+GW, 10 μM added to both sides), capsaicin (+Caps, 1 μM added both sides) or BIBO3304 (+BIBO, 300 nM basolaterally). A significant difference between control and BIBO-pretreated OEA responses is shown, ** $P \leq 0.01$; *** $P \leq 0.001$.

(D) To the left, PYY released from WT colon mucosa at time (T) 0 or after 90 min incubation with either vehicle (+Veh, 1% DMSO) or tolbutamide (1mM; * $P \leq 0.05$ compared with T=0). To the right, total tissue PYY levels are shown after 90 min incubation of WT colon mucosa (± tolbutamide) compared with PYY levels from each genotype. Differences between peptide levels in null tissues compared with WT controls are shown, *** $P \leq 0.001$. Each bar is the mean ± SEM with observation numbers shown in parentheses.
